# PKMζ Maintains Spatial, Instrumental, and Classically Conditioned Long-Term Memories

**DOI:** 10.1371/journal.pbio.0060318

**Published:** 2008-12-23

**Authors:** Peter Serrano, Eugenia L Friedman, Jana Kenney, Stephen M Taubenfeld, Joshua M Zimmerman, John Hanna, Cristina Alberini, Ann E Kelley, Stephen Maren, Jerry W Rudy, Jerry C. P Yin, Todd C Sacktor, André A Fenton

**Affiliations:** 1 Department of Physiology and Pharmacology, The Robert F. Furchgott Center for Neural and Behavioral Science, State University of New York, Brooklyn, New York, United States of America; 2 Department of Psychiatry, University of Wisconsin-Madison Medical School, Madison, Wisconsin, United States of America; 3 Neuroscience Training Program, University of Wisconsin-Madison Medical School, Madison, Wisconsin, United States of America; 4 Institute of Physiology, Academy of Sciences of the Czech Republic, Prague, Czech Republic; 5 Fishberg Department of Neuroscience, Mount Sinai School of Medicine, Icahn Medical Institute, New York, New York, United States of America; 6 Neuroscience Program, University of Michigan, Ann Arbor, Michigan, United States of America; 7 Department of Psychology, University of Michigan, Ann Arbor, Michigan, United States of America; 8 Department of Psychology, Center for Neuroscience, University of Colorado, Boulder, Colorado, United States of America; 9 Department of Genetics, University of Wisconsin-Madison Medical School, Madison, Wisconsin, United States of America; 10 Department of Neurology, The Robert F. Furchgott Center for Neural and Behavioral Science, State University of New York, Brooklyn, New York, United States of America; National Institutes of Health, United States of America

## Abstract

How long-term memories are stored is a fundamental question in neuroscience. The first molecular mechanism for long-term memory storage in the brain was recently identified as the persistent action of protein kinase Mzeta (PKMζ), an autonomously active atypical protein kinase C (PKC) isoform critical for the maintenance of long-term potentiation (LTP). PKMζ maintains aversively conditioned associations, but what general form of information the kinase encodes in the brain is unknown. We first confirmed the specificity of the action of zeta inhibitory peptide (ZIP) by disrupting long-term memory for active place avoidance with chelerythrine, a second inhibitor of PKMζ activity. We then examined, using ZIP, the effect of PKMζ inhibition in dorsal hippocampus (DH) and basolateral amygdala (BLA) on retention of 1-d-old information acquired in the radial arm maze, water maze, inhibitory avoidance, and contextual and cued fear conditioning paradigms. In the DH, PKMζ inhibition selectively disrupted retention of information for spatial reference, but not spatial working memory in the radial arm maze, and precise, but not coarse spatial information in the water maze. Thus retention of accurate spatial, but not procedural and contextual information required PKMζ activity. Similarly, PKMζ inhibition in the hippocampus did not affect contextual information after fear conditioning. In contrast, PKMζ inhibition in the BLA impaired retention of classical conditioned stimulus–unconditioned stimulus (CS-US) associations for both contextual and auditory fear, as well as instrumentally conditioned inhibitory avoidance. PKMζ inhibition had no effect on postshock freezing, indicating fear expression mediated by the BLA remained intact. Thus, persistent PKMζ activity is a general mechanism for both appetitively and aversively motivated retention of specific, accurate learned information, but is not required for processing contextual, imprecise, or procedural information.

## Author Summary

How long-term memories are stored as physical traces in the brain is a fundamental question in neuroscience. Recently, we discovered the first molecular mechanism of long-term memory storage. We showed that unpleasant memories are stored by the persistent action of an enzyme, a form of protein kinase C, termed PKMζ, because these memories can be rapidly erased by injecting a PKMζ inhibitor into the brain. But are all forms of memory and information in the brain stored by PKMζ? Here, we first confirmed with a second inhibitor of PKMζ that unpleasant long-term memories in the hippocampus, a region of the brain critical for storing spatial information, are rapidly erased. We then examined other memories stored in the hippocampus and the basolateral amygdala, another region critical for emotional memories. We tested memories for specific places, both unpleasant and rewarding, memories for general background information, associations between a sound and a fearful event, like that studied by Pavlov, and memories for performing a specific action. We found that PKMζ stores specific associations, both unpleasant and rewarding, for places, events, and actions, and is thus a general mechanism for memory storage in the brain.

## Abstract 3

The persistent activity of a brain enzyme, PKMζ, stores specific associations, both unpleasant and rewarding, for places, events, and actions, and is thus a general mechanism for memory storage.

## Introduction

Although the molecular mechanisms of initial memory consolidation have been extensively studied, little is known about the mechanism of persistent memory storage [1]. Recently, however, the persistent phosphorylation by the autonomously active protein kinase C (PKC) isoform, protein kinase Mzeta (PKMζ), has been shown to be critical for the maintenance of aversive long-term memories, specifically, place avoidance in the hippocampus [2] and conditioned taste aversion in the neocortex [3].

PKMζ was initially identified as a persistently active kinase that is both necessary and sufficient for the maintenance of long-term potentiation (LTP) [4,5]. PKMζ is a persistently active kinase because of its unique structure [4]. Most PKC isoforms consist of an N-terminal regulatory domain, which contains second messenger-binding sites and an autoinhibitory pseudosubstrate sequence, and a C-terminal catalytic domain [6]. Under basal conditions, the pseudosubstrate interacts with the catalytic domain and maintains the enzyme in an autoinhibited resting state. Second messengers, such as diacylglycerol or Ca^2+^, can then activate full-length PKCs by binding to the regulatory domain, causing a conformational change that releases the autoinhibition. PKMζ, in contrast, is an independent PKCζ catalytic domain, which, lacking autoinhibition from a regulatory domain, is autonomously active. In the brain, PKMζ is generated by an internal promoter within the *PKCζ* gene, which produces a PKMζ mRNA that encodes only the ζ catalytic domain [7]. During LTP, tetanic stimulation induces de novo synthesis of PKMζ, increasing the amount of the persistently active kinase [7,8]. The persistent PKMζ activity is critical for maintaining enhanced synaptic transmission, because inhibition by the cell-permeable zeta inhibitory peptide (ZIP), which mimics the pseudosubstrate of the missing PKCζ regulatory domain, reverses synaptic potentiation in the hippocampus when applied up to 1 d after LTP induction [2]. The effect is specific to potentiated synapses because the same dose of ZIP does not affect baseline synaptic transmission [2].

In parallel studies, this dose of ZIP eliminated the retention of long-term memory, but not short-term memory, for place avoidance in the hippocampus [2] and disrupted the storage, but not acquisition, of conditioned taste aversion in the insular neocortex [3]. Despite affecting retention of these aversively conditioned long-term memories, PKMζ inhibition had no effect on taste familiarity, although it is not known whether memory supporting taste familiarity is stored in the insula [3]. Thus, whether the persistent activity of PKMζ maintains all information in a brain region is a critical open question.

To address this issue, we studied a battery of conditioned behaviors that require either the dorsal hippocampus (DH) or the basolateral amygdala (BLA) for memory retention, as previously determined by posttraining ablation studies. To compare results across a range of types of long-term memory induced by widely different behavioral paradigms, 1 d after the completion of training to acquire long-term memory, we injected the standard dose of ZIP that locally reverses 1-d-old in vivo LTP without affecting baseline synaptic transmission [2].

## Results

### Spatial Memory

**Figure 1:**
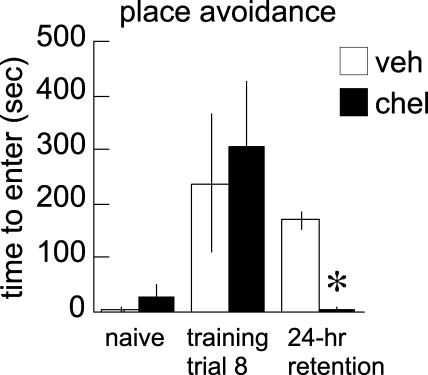
Chelerythrine in DH Disrupts Place Avoidance Memory Naive rats rapidly entered the shock zone on the first training trial but learned to avoid the location for several minutes by the eighth training trial. Chelerythrine (chel) or vehicle (veh) was injected in the DH 2 h before testing retention of 24-h memory. Chelerythrine, but not saline, eliminated retention of the memory, causing avoidance to drop to the level of when the rats were naive (F_2,21_ = 14.2; *p* = 0.0001; an asterisk (*) indicates *p* < 0.05, chel relative to veh). All data are presented as averages, with error bars indicating standard errors of the mean.

Previous studies in the DH of place avoidance memory had shown that the PKMζ inhibitor ZIP, but not the conventional and novel PKC isoform inhibitor staurosporine, which does not effectively inhibit PKMζ, caused a selective loss of long-term memory retention [2]. The only other potent PKMζ inhibitor to have been characterized is chelerythrine, a benzophenanthridine alkaloid rather than a pseudosubstrate peptide like ZIP, and a general inhibitor of the catalytic domain of PKCs that strongly inhibits PKM forms [5]. We therefore examined the effect of chelerythrine on long-term memory retention of active place avoidance. On the first trial, rats entered the shock zone within seconds, but with training, the animals learned to avoid the shock zone for several minutes (Figure 1). Twenty-two hours later, chelerythrine or vehicle was injected into both hippocampi, and retention of the avoidance memory was tested 2 h after the injection, as previously described for ZIP [2]. Rats showed excellent retention following injections of the control solution, avoiding the shock zone for several minutes (Figure 1). However, after injections of chelerythrine, the rats once again rapidly entered the shock zone within seconds (Figure 1).

Chelerythrine did not prevent acquiring or expressing short-term memory for active place avoidance. Immediately after the 24-h retention test, the shock was turned on for 10 min and then turned off [2]. Turning on the shock improved avoidance in the vehicle-injected rats even further. Thus, during the long-term memory testing, the animals spent only 7.9 ± 0.7% of their time in the shock zone, significantly less than 16.7%, the level of chance (*t*_3_ = 12.0; *p* = 0.001), and then after the single training session, they spent even less time in the shock zone (0.44 ± 0.44%; *t*_3_ = 37; *p* < 0.0001 compared to chance). During the long-term memory testing, the animals injected with chelerythrine showed no long-term memory retention, as expected, spending time in the shock zone at the level of chance (16.4 ± 1.1%, *t*_4_ = 0.3; *p* = 0.7), but then these animals avoided the shock zone after the single training session, spending only 9.3 ± 1.1% of the session in the shock zone after the shock was turned off (*t*_4_ = 6.5; *p* = 0.003 compared to chance). Thus, the action of chelerythrine on both long-term and short-term memory retention was indistinguishable from the action of ZIP [2]. In subsequent experiments, we used ZIP, the more specific of the two drugs, and controlled for nonspecific effects of the peptide in each of the conditioned behaviors with the scrambled, inactive version of ZIP (scr-ZIP) [2].

**Figure 2:**
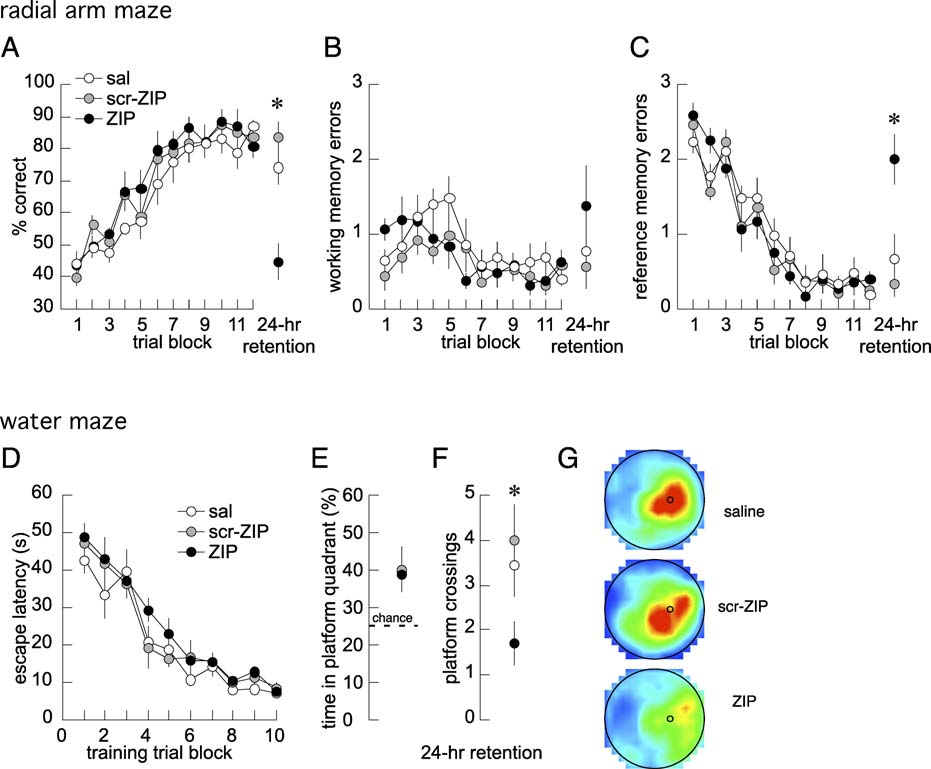
ZIP in DH Disrupts Spatial Memory (A) Performance of the eight-arm radial maze task. Learning across 6 d (ten trials per day) was followed by a single retention trial after a 24-h interval. Two hours before the retention trial, each rat received a bilateral DH injection of either saline (sal, *n* = 9), the control peptide (scr-ZIP, *n* = 9), or ZIP (*n* = 8). The ZIP injection impaired overall performance ([A]; *F*_2,23_ = 14.80; *p* = 10^−5^) by increasing reference memory errors ([C]; *F*_2,23_ = 9.30; *p* = 0.001) without increasing working memory errors ([B]; *F*_2,23_ = 1.16; *p* = 0.33). (D–G) Performance of the water maze task (D) during training (two four-trial blocks per day) and (E–G) during the unreinforced swim retention test after a 24-h interval. Each rat received a bilateral DH infusion of saline (*n* = 7), scr-ZIP (*n* = 7), or ZIP (*n* = 10) 2 h before the retention test. (E) Percent time in the target quadrant, (F) number of times the position of the escape platform was crossed, and (G) the color-coded time-in-location map for each treatment group during the retention trial. The same blue-to-red scale is used for each map, where the minimum time in the peak, red category is 0.9 s. ZIP impaired retention of spatial accuracy (*F*_2,21_ = 3.96; *p* = 0.03), but not the spatial search procedure (*F*_2,21_ = 2.08; *p* = 0.15). All data are presented as averages, with error bars indicating standard errors of the mean. An asterisk (*) indicates *p* < 0.05, ZIP relative to saline and scr-ZIP.

We then examined whether appetitively conditioned spatial information was maintained by the same mechanism as aversively conditioned spatial information in the DH by examining the effect of ZIP on conditioned behavior in the eight-arm radial maze. Rats learned the task, and after six trial blocks (3 d), performance was asymptotic and optimal for an additional six trial blocks (days 4–6; Figure 2A–2C). On day 7, bilateral DH injections of the control compounds, saline or scr-ZIP, did not alter performance during testing that began 2 h later. In contrast, injections of ZIP caused the number of correct choices to drop to the level of naive rats (Figure 2A–2C). The deficit could not be attributed to an increase in working memory errors (Figure 2B; *p* = 0.33), but was due to a specific increase in spatial reference memory errors (Figure 2C; *p* = 0.001).

To further characterize these reference memory errors, we examined the errors made by the ZIP-injected rats when there was only one correct choice remaining, i.e., after three of the four baited arms had been chosen. Of 21 such errors, ten were to an arm adjacent to the correct arm, and six, three, and two were to arms that were two, three, or four arms away from the correct arm, respectively. After accounting for the fact that there was only a single arm four arms away from the correct arm, and two arms for the other categories, there was a significant effect of where the errors were distributed (*F*_3,28_ = 3.1; *p* = 0.04). Thus, ZIP impaired spatial reference memory, possibly by impairing spatial accuracy, but because the rats foraged appropriately on the maze and continued to use the win-shift strategy that requires working memory, the memory for the general contextual and procedural aspects of the task appeared unaffected.

In the active place avoidance task, ZIP had a persistent effect on long-term memory retention [2]. To examine whether impairment in the radial arm maze was also persistent, 2 wk after the ZIP injection, the animals were reexamined with a single training trial. The rats that had been injected with saline 2 wk earlier showed excellent memory retention (96.7 ± 6.5% correct choices), whereas the rats that had been injected with ZIP made fewer correct choices (59.1 ± 6.7%), indicating they were still impaired (*t*_11_ = 8.6; *p* < 0.0001).

We next examined whether spatial reference memory in the water maze is also maintained by PKMζ in the hippocampus. Rats learned the location of the escape platform during 5 d of training (Figure 2D). After injections with the control solutions, saline or scr-ZIP, 2 h before the probe test on day 6, the rats repeatedly crossed the platform location and concentrated their search in the correct quadrant (Figure 2E–2G). In contrast, ZIP injections diminished the accuracy of searching. Although the ZIP-injected rats concentrated their swim time in the correct quadrant of the pool (Figure 2E and 2G; *p* = 0.15), they crossed the platform location fewer times than the rats injected with saline or the control compound (Figure 2F and 2G; *p* = 0.03). These differences were not due to changes in the total distance the rats swam (saline [sal] = 15.2 ± 0.4 m; scr-ZIP = 16.3 ± 0.5 m; and ZIP = 16.3 ± 0.5 m; *F*_2,21_ = 1.56; *p* = 0.2) or the average swim speed calculated every 2 s (sal = 25.3 ± 0.7 cm/s; scr-ZIP = 27.2 ± 0.8 cm/s; and ZIP = 27.2 ± 0.9 cm/s; *F*_2,21_ = 1.56; *p* = 0.2). The amnesia induced by ZIP persisted, because on day 29, the rats that had been injected with scr-ZIP 23 d earlier, took 18.4 ± 3.5 s to find the platform on the first trial, in contrast to the rats that had previously received ZIP injections, which took significantly longer, 43.4 ± 9.4 s (*n*'s = 4; *t*_7_ = 2.6; one-tailed *p* = 0.04). These results indicate that PKMζ in DH maintains the precise spatial information that is needed for accurate localization, but not the global spatial information or the contextual information that is necessary for the spatial search strategy.

### Classically Conditioned Fear Memory

**Figure 3:**
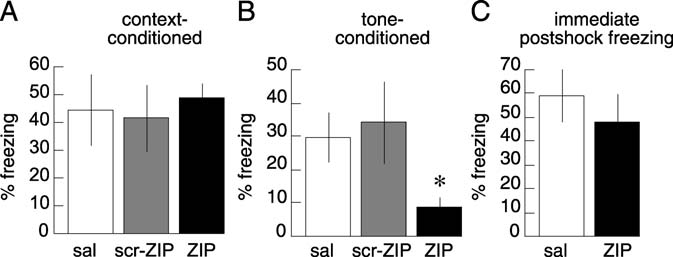
ZIP in BLA Disrupts Classically Conditioned Fear Memory (A) Retention of context-conditioned fear 26 h after bilateral DH injection of saline (sal, *n* = 4), inactive (scr-ZIP, *n* = 7), or active ZIP (*n* = 6). ZIP did not impair retention of contextual fear (*F*_2,14_ = 0.15; *p* = 0.86). (B) Retention of tone-conditioned fear after 22-h posttraining bilateral BLA injections. Retention was tested 2 h (sal, *n* = 6; scr-ZIP *n* = 3; ZIP *n* = 10) or 24 h (sal, *n* = 5; scr-ZIP *n* = 4; ZIP *n* = 8) after the injection. ZIP impaired retention of tone-conditioned fear (*F*_2,33_ = 4.93; *p* = 0.01). (C) Immediate postshock freezing after bilateral BLA injections. Fear was tested 5 min (sal, *n* = 4; ZIP, *n* = 4) or 120 min (sal, *n* = 5; ZIP, *n* = 5) after the injections. ZIP did not affect immediate postshock freezing, indicating the expression of fear was intact (*F*_1,16_ = 0.58; *p* = 0.46). All data are presented as averages, with error bars indicating standard errors of the mean.

We then tested whether persistent PKMζ activity in the hippocampus maintains conditioned-fear responses to context. ZIP injections into the DH 22 h after context/tone-shock pairing failed to alter contextual freezing tested 1 d later (Figure 3A; *p* = 0.86). Although the DH may have a role in some tone-fear paradigms [9,10], its role in long-term storage of tone-fear associations is uncertain [10–12]. ZIP in the DH also did not impair tone-associated fear tested in a novel chamber 3 d after the infusions (unpublished data). In additional experiments, decreasing the number of shocks from five to one, eliminating the tone during conditioning with a single shock, and bilaterally injecting into both dorsal and ventral hippocampi 1 d after conditioning failed to reveal an effect of ZIP on contextual fear (sal, 86 ± 11% freezing; ZIP, 83 ± 9% freezing; *F*_1,14_ = 0.32; *p* = 0.9).

Together, these results in the DH indicate that PKMζ selectively maintains precise learned associations for locations, but not associations to imprecise spatial or background contextual stimuli. We therefore tested whether PKMζ maintains the fear-mediated CS-US associations thought to be stored in the BLA. Rats received a single tone-shock pairing trial and 22 h later were injected with saline, scr-ZIP, or ZIP. The saline- and scr-ZIP–treated rats expressed normal conditioned fear 2 h and 24 h after the injection, but the ZIP-injected rats showed impaired conditioned freezing at both retention delays. The results of the two retention delays were indistinguishable and therefore analyzed together. The effect of ZIP was different from that of the control solutions (Figure 3B; *p* = 0.01). In separate animals (*n* = 8 for each group), the ZIP injections into the BLA 24 h after context/tone-shock pairing attenuated both contextual freezing tested 1 d later and tone-associated fear tested in a novel chamber 3 h after the context test (freezing to context: sal, 82 ± 4.5%; ZIP, 48 ± 6.7%; *F*_1,14_ =18.2, *p* < 0.001; freezing to tone: sal, 61 ± 12%; ZIP, 8 ± 3%; *F*_1,14_ = 20.3, *p* < 0.001). In parallel locomotion experiments, ZIP infusion did not induce hyperactivity measured by beam crossings during 1 h (scr-ZIP, average = 146.4 ± 15.7, *n* = 4; ZIP, average = 197.2 ± 35.8, *n* = 5; *F*_1,7_ = 1.7, *p* = 0.23). Thus, in contrast to the DH, ZIP injection into the BLA impairs retention of conditioned-fear behavior.

Ablation of the BLA as well as the adjacent central nucleus of the amygdala attenuates freezing to the shock itself [13–15], which may confound the interpretation of whether information is stored in the BLA, or instead, whether the BLA is required for the expression of a fear association that is stored elsewhere. We therefore tested whether injecting ZIP into the BLA affected the expression of fear immediately after a shock. ZIP or saline was infused into the BLA 5 min or 2 h prior to testing immediate postshock freezing. ZIP did not affect immediate postshock freezing at either time point (Figure 3C; data from both time points combined, *p* = 0.46). Because ZIP in the BLA did not alter the ability to express fear, but attenuated conditioned fear, we conclude that persistent PKMζ activity in the BLA maintains the information that is required for fear associations, but not the function of the BLA in expressing fear.

### Instrumentally Conditioned Memory

**Figure 4:**
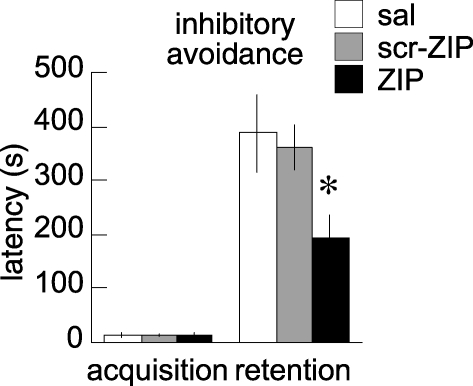
ZIP in BLA Disrupts Inhibitory Avoidance Memory Latency to enter the dark compartment during the acquisition and retention phases of inhibitory avoidance. Retention was tested 24 h after acquisition, 2 h after the bilateral BLA injections (sal, *n* = 7; scr-ZIP, *n* = 15; and ZIP, *n* = 16). The latency to enter the dark compartment was similar across groups during acquisition, but not during retention. The interaction between group and phase of the inhibitory avoidance task was significant (*F*_2,35_ = 4.28; *p* = 0.02). ZIP impaired retention of inhibitory avoidance compared to the animals treated with saline and scr-ZIP, which were indistinguishable. The asterisk (*) indicates *p* < 0.01, ZIP relative to saline and scr-ZIP. All data are presented as averages, with error bars indicating standard errors of the mean.

We then tested whether other forms of memory that depend on the BLA also require persistent PKMζ activity for maintenance. Injecting ZIP into the BLA 22 h after inhibitory avoidance conditioning impaired retention of the conditioned response that was tested 2 h later (Figure 4; *p* < 0.01). Two weeks later, the impairment persisted (latency to enter the dark compartment: *n*'s = 4; scr-ZIP = 297 ± 104 s; ZIP = 79 ± 50 s; *t*_7_ = 2.5; one-tailed *p* < 0.05). Thus, long-term memories for both classically conditioned fear and instrumentally conditioned inhibitory avoidance depend upon persistent PKMζ activity in the BLA.

## Discussion

We find that site-specific inhibition of PKMζ impairs the retention of specific, accurate associations in multiple tasks in different brain regions, regardless of positive or negative reinforcement, and thus the persistence of PKMζ activity is a general molecular mechanism for the maintenance of memory. This mechanism is specific for sustaining accurate learned associations because inhibition of the kinase did not affect the expression of imprecise, contextual, and procedural information that depends upon the functioning of the brain regions in which the associations were stored. PKMζ inhibition thus contrasts with permanent or temporary lesions, which affect both types of information. Because ZIP specifically reverses information stored in synapses by late-LTP maintenance and does not affect baseline synaptic transmission in the hippocampus [2,5,16,17], these results suggest that the physiological function of late-LTP–like plasticity may also be selectively important for storing specific accurate information. Although future work will be required to examine whether PKMζ maintains late LTP in the BLA as well the hippocampus, in all the tasks, ZIP produced a persistent loss of long-term memory, consistent with previous results of an effect on memory storage [2,3], although the possibility of an as yet undiscovered role in information retrieval cannot be ruled out.

In our study, we first replicated the main finding of Pastalkova et al. [2], using a second inhibitor of PKMζ, chelerythrine. We found that the drug, the only other potent inhibitor of PKMζ activity known, produces the identical rapid impairment of long-term memory retention but sparing of short-term memory. Although chelerythrine affects all PKC isoforms at high doses [5], (1) ZIP does not affect conventional and novel PKCs [5], and inhibition of these other PKCs does not affect memory retention [2]; and (2) the common target of the two inhibitors is PKMζ, and both agents cause the same pattern of amnesia. The possibility that ZIP might inhibit another as yet unidentified protein kinase or some other process cannot be excluded [18]; however, such an effect would require the specific sequence of amino acids in ZIP that inhibits PKMζ activity, because the effect of the scrambled version of the peptide was indistinguishable from saline in all of the behaviors examined in this study.

In the DH, persistent PKMζ activity was shown to specifically maintain memories for precise spatial locations, but not imprecise spatial, contextual, or procedural information. Thus, the same injections of ZIP resulted in the loss of information supporting accurate spatial reference memory in the eight-arm radial maze task (Figure 2C), but no effect on the ability to use working memory to do the win-shift foraging procedure (Figure 2B). Likewise, the effect of ZIP injection in the water maze task was the elimination of information supporting accurate spatial navigation (Figure 2F), whereas information needed for the general place response to search in the platform quadrant of the pool was spared (Figure 2E and 2G). Thus, the general recognition of the contextual and the procedural aspects of these tasks appeared unaffected by ZIP. Indeed, the equivalent ZIP injection in the DH did not impair context-associated fear at all (Figure 3A), whereas injection of the inhibitor in the BLA disrupted the conditioned response. It is possible that regions of the hippocampus not affected by the ZIP injections contributed to the sparing of contextual aspects of memory [19]; however, we consider this unlikely because simultaneous ZIP injections in dorsal and ventral hippocampi also did not affect contextual fear. Thus, long-term information stored within the DH by PKMζ activity appears to be required for fine, accurate spatial reckoning or precise discrimination between related memories of location, as between the arms in the radial maze. These findings are consistent with the complete loss of the ability to perform active place avoidance on a rotating disk following PKMζ inhibition (Figure 1 and [2]). In this task, the ability to discriminate between specific memories of shock locations with respect to the room and shock locations on the rotating disk is essential for avoiding the stationary shock zone [20,21].

The ability of ZIP to disrupt specific types of stored information in the DH while leaving other information intact may be due to its ability to specifically reverse late-LTP maintenance in the hippocampus, but not other forms of neural plasticity that can store information [22]. For example, PKMζ activity in the DH is not required for working memory in a radial arm maze, which appears to be mediated by transient early LTP [23,24] that is not maintained by PKMζ [16,17]. Long-term memories encoding coarser-grained spatial positions or context may be mediated by forms of long-term synaptic plasticity that might not be maintained by PKMζ, such as long-term depression [17,22] or perhaps, changes in neural excitability [25–27].

Alternatively, memories unaffected by ZIP in the DH may be stored elsewhere but require an intact hippocampus for processing or retrieving the information rather than storing it. Indeed, the specific effect of ZIP on fine, but not coarse-grained spatial information in the DH is consistent with the recent discovery that grid cells of the medial entorhinal cortex, which is the main input to the hippocampus, provide sufficient contextual and place information for spatial navigation based on distal landmarks [28–30]. Thus extrahippocampal regions appear to encode sufficient spatial information for the rat to recognize its environment and general location. Prior work with ablation or inactivation of the hippocampus would have interrupted the projection loops of this spatial information from the superficial layers of the entorhinal cortex through the subfields of the hippocampus and back out to the deep layers of the entorhinal cortex. In contrast, transmission of information through these circuits may not have been disrupted by PKMζ inhibition because ZIP has no effect on baseline synaptic transmission in hippocampal slices or in vivo [2,5,16,17].

In the BLA, both specific instrumentally conditioned associations for inhibitory avoidance and classically conditioned associations for fear were impaired by PKMζ inhibition. The impairment was specific to long-term memory because the PKMζ inhibition did not induce hyperlocomotion or disrupt the expression of fear to a recent shock, as observed, for example, with ablations of the BLA [13–15, 31]. Thus, specific fear memories are maintained in the BLA by persistent PKMζ activity, which may be distinct from the BLA's role in modulating aversively motivated information stored elsewhere [32,33].

Lastly, the characterization of the forms of information stored in the brain by PKMζ may have clinical implications for disorders thought to be mediated by excessive memory retention or LTP-like plasticity, such as posttraumatic stress, phobias, and addictions. Previous studies have indicated that PKMζ inhibition by ZIP in the hippocampus and neocortex erases long-term memories encoded even weeks prior to injection [2,3]; therefore, further study will ultimately be required to identify a method by which PKMζ inhibition might target specific memories, perhaps by examining the role of PKMζ during reconsolidation after memory reactivation [34,35]. As an enabling first step, however, our current findings suggest that PKMζ inhibition disrupts the retention of specific, precise information stored in a brain region, but spares the region's processing functions such as relaying information or performing computations on information stored elsewhere. Thus, not all memories and functions previously ascribed to a brain region will be lost by site-specific PKMζ inhibition, but discrete pathophysiological associations induced by both fearful and rewarding experiences may.

## Materials and Methods

**Figure 5:**
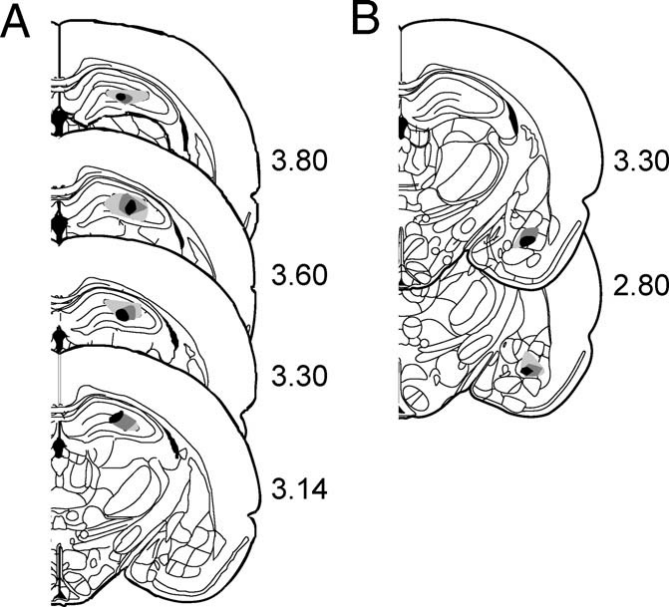
Characterization of the Infusion Sites in the (A) DH and (B) BLA Infusion sites from a subset of the animals are plotted on schematics of the brain at the indicated distance in millimeters posterior to bregma. The one third of the injection sites closest to the centroid location at each level fell within the black region. Two thirds fell within the medium-gray region, and all injections fell within the light-gray region.

Adult male rats were implanted with cannulae for intracranial infusion in the DH or BLA (Figure 5). The rats were first trained to acquire a robust long-term memory in a particular behavioral paradigm. A day later, we tested whether bilateral inhibition of PKMζ impaired memory retention, by injecting a PKMζ inhibitor or a control solution [2]. All experimental methods have been published and are briefly described here. The procedures were approved by the local institutional animal care and use committee in compliance with the National Institutes of Health and federal guidelines.

### Intracranial infusion.

Each rat was deeply anesthetized (Nembutal >50 mg/kg intraperitoneally [i.p.] or ketamine/xylazine mixture 100/10 mg/kg i.p.) and mounted in a stereotaxic frame to drill bilateral holes in the skull for a pair of 22-ga infusion guide cannulae. The guide tips were at least 1 mm above the infusion target. Bone screws and dental cement secured the cannulae to the skull. Each rat was handled for at least 5 d to habituate it to the experimenter prior to training. Training began at least a week after surgery. The rats were habituated to the infusion procedure by mock saline infusions at least a day before the experimental solutions were infused. After the task was well learned, each rat received a bilateral intracranial infusion (1 μl/side) of one of three experimental solutions: the myristoylated peptide PKMζ inhibitor ZIP (10 nmol/μl saline; QCB and University Wisconsin Biotech peptide synthesis facility), the control myristoylated peptide, scr-ZIP, comprising a scrambled sequence of the same amino acids as ZIP (10 nmol/μl saline; QCB), or saline [2]. Memory retention was tested 2 h after the infusion, unless stated otherwise. The infusion target in the DH was 3.8 mm posterior, 2.5 mm lateral, and 3.5 mm ventral to bregma. In the experiment to examine the effect of simultaneous PKMζ inhibition of both the dorsal and the ventral hippocampi, the dorsal coordinates were 2.5 mm posterior, 2.4 mm lateral, and 3.0 mm ventral to bregma, and the ventral coordinates were 5.6 mm posterior, 5.0 mm lateral, and 6.0 mm ventral to bregma. The coordinates for bilateral infusion into the BLA were 2.8 mm posterior, 4.8 mm lateral, and 5.8 mm ventral to bregma. After behavioral testing, the rats were sacrificed by anesthetic overdose, then transcardially perfused with saline followed by 10% formalin. The brains were removed, postfixed in 10% sucrose-10% formalin solution, sectioned, then stained with cresyl violet, and examined by light microscopy to estimate the injection site (Figure 5).

### Active place avoidance.

Active place avoidance memory is a rapidly acquired form of spatial memory, the long-term retention of which is disrupted by bilateral hippocampal inactivation [36]. The first demonstrations that PKMζ maintains long-term memory used ZIP in DH to eliminate 1-d-old place avoidance memories [2]. We used the same training protocol to test whether chelerythrine, another PKMζ inhibitor, also eliminates long-term place avoidance memory.

The place avoidance procedures have been described in detail [2,20]. Briefly, the rat is placed on an 82-cm–diameter metal disk that is elevated 78 cm from the floor and rotates at 1 rpm within a room with numerous visual landmarks off of the disk. Prior to training, the rat is implanted with a subcutaneous shock electrode between the shoulders, through which a constant current (0.3 mA, 60 Hz, 500 ms) electrical foot shock is delivered whenever the rat enters an unmarked shock zone. The impedance between the shock electrode and the skin is approximately 1,000 times less than the impedance between the rat's feet and the metal disk, which is grounded, so the major voltage drop is across the feet. The shock zone is an unmarked 60° sector that is defined by distal visual landmarks in the room. The location of the rat is determined from an overhead television camera each 33 ms by a PC-controlled tracking system (Bio-Signal Group). When the system detects the rat in the shock zone, the shock is delivered and repeated every 1,500 ms until the rat leaves the shock zone.

Place avoidance training begins with a pretraining trial. The rat is placed on the rotating disk to explore the environment with the shock turned off for 10 min. After resting in the home cage for 10 min, the rat receives eight training trials with the shock turned on. There is a 10-min rest in the home cage between trials. Twenty-two hours after training, the rat was injected in both hippocampi with either chelerythrine (*n* = 5; 10 nmol in 50% DMSO-saline) or the vehicle (*n* = 4), and 2 h later, retention of the 24-h place avoidance memory was tested by returning the rat to the rotating disk with the shock off. The time to first enter 9th shock zone and the percent time in the shock zone estimated retention of memory. Immediately following the retention test for long-term memory, short-term memory was assessed. Short-term memory is established by turning on the shock for 10 min, and then retention is tested during a 10-min test period with the shock off.

### Eight-arm radial maze.

Spatial reference memory is distinguished from spatial working memory in the eight-arm radial maze because in reference memory, information about which arm locations are consistently baited is valid across trials, whereas working memory requires spatial information for which arm locations were visited within a trial, information that is only useful for the specific trial. We used the standard four-arms baited, four-arms unbaited task variant [37]. In this task, lesions of the hippocampus increase working memory errors, but not reference memory errors [37]. This basic result [38,39] contrasts, however, with many studies indicating that the hippocampus is critical for spatial reference memory in water maze tasks and other tests of spatial reference memory [40].

Detailed surgical methods are reported in [20,21]. The rats were food deprived to 85%–90% of their free-feeding weight prior to training on the eight-arm radial maze. The maze was 220 cm in diameter with a 60-cm–diameter central platform. Each arm was 16-cm wide and radiated 80 cm from the center. The maze was wiped with 70% ethanol between trials and rotated 90° every day to discourage the use of internal maze cues. The day before formal training began, each rat received two 10-min shaping trials with all arms baited by placing approximately 0.05 g of a sweetened oatmeal cereal mash (Maypo; International Home Foods) in the sunken food well at the end of each arm. Two rats were on the maze for the first shaping trial; and 1 h later, each rat received a second shaping trail by itself. On training trials, four arms were baited, and the food cups at the ends of the unbaited arms had inaccessible mash to control for odor cues. The locations of baited and unbaited arms were constant for a subject and balanced across subjects. There were ten training trials on each day. The rat was confined to the center of the maze by a large, overturned transparent bowl prior to each trial. Once released, the rat was free to forage until it consumed all the accessible food, or until 3 min had elapsed. Entry to an arm was scored when the rat crossed the halfway point of an arm. A trial was scored for correct entries, reference memory errors (visits to unbaited arms), and working memory errors (return visits to an arm) [41]. Training continued for 6 d (60 trials) to establish a strong memory. The next day, 24 h after training ceased, a single reinforced trial was given to test memory. Two hours prior to the memory test, each rat received a bilateral DH infusion of saline, scr-ZIP, or ZIP.

### Water maze.

We used a standard version of the water maze to assess spatial reference memory. The training protocol establishes a hippocampus-dependent memory, which can be demonstrated on day 6 by an inability to localize searching for the platform on a probe trial following hippocampal inactivation [20].

The consensus is that the DH is important for spatial reference memory in the water maze, but whether the DH is crucial for storing this spatial reference memory is controversial. Although tetanic stimulation to saturate potentiation of synaptic transmission, pharmacological blockade of *N*-methyl-D-aspartate receptors, and permanent and functional lesions of the DH all impair learning and memory of the escape location [20,42,43], the impairment is absent in rats that had learned the water maze procedure, but not the particular escape location, prior to the amnestic intervention [44–47].

Detailed methods were reported [20]. The rats were trained in a 1.83-m diameter circular pool filled with 40-cm deep 21–22 °C opaque water. The rats were trained to find a circular, 10-cm–diameter clear Perspex platform that was submerged 1 cm below the water surface halfway between the center of the pool and the wall along the 0° radius. A rat was released in pseudorandom order from one of four equally separated locations along the wall. Each release location was used once in a four-trial training block. The path of the rat was automatically tracked with an overhead television camera (iTrack; Bio-Signal Group). Software (TrackAnalysis and TrackExplorer; Bio-Signal Group) calculated the latency to escape onto the platform, the time spent in each quadrant centered at 0°, 90°, 180°, and 270°, the time in each 11.5-cm–square region of the pool, and each 2 s, the rat's swim speed. Acquisition training lasted 5 d (two blocks/day). On day 6, 22 h after the last training, the rats were injected with saline, scr-ZIP, or ZIP, and 2 h after that, retention of the long-term place memory was tested by a probe trial with the escape platform removed. On this probe, the rat was placed in the center of the pool, and where it spent its time was measured for 1 min.

### Fear conditioning.

*Contextual fear.* Lesions of the DH disrupt the general, contextual component of the learned fear response in contextual conditioning paradigms. We conditioned rats in a standard, combined context and tone-conditioned fear protocol in which the rat received five shocks [9]. Following this training, long-term retention of contextual, but not tone fear is impaired by posttraining DH lesions [9,48]. We also examined two other context-conditioning protocols in which only a single shock was administered. In one protocol, the shock was paired with a tone; in the other, the tone was not presented.

The procedures have been described in detail [49]. Aluminum and Plexiglas conditioning chambers housed in sound-attenuating cabinets were used. The floor of each chamber was made of parallel rods that could deliver pole-scrambled, constant current foot shock (1.0 mA, 2 s). The chambers were contextually distinct. “Context A” (used for conditioning and context retention testing), had working ventilation fans that produced background noise (65 dB). The chamber lights and room lights illuminated the space because the sound-attenuating chest doors were open. The chambers were cleaned with a 1% ammonium hydroxide solution, which covered the surface under the shock floor. The chest doors were closed for “Context B” (used for tone retention testing), and fluorescent red light provided illumination. The ventilation fans were inactive, and the chambers were cleaned with a 1% acetic acid solution, which covered the pan below the shock floor.

Movement in each chamber was monitored using load cell inputs to a 5-Hz analog-to-digital converter calibrated to activity values between 0 and 100. Freezing behavior was automatically detected as inactivity (activity value <10) during at least 1 s (Threshold Activity software; Med-Associates).

Each rat was placed in a conditioning chamber for training, and after 3 min, five tone (2 kHz, 80 dB, 10 s) shock (1.0 mA, 2 s) pairings were delivered (70-s intertrial interval). The tone coterminated with the shock. Twenty-two hours after training, both dorsal hippocampi were infused with the saline, scr-ZIP, or ZIP solutions. Twenty-six hours after the infusions, long-term retention of contextual fear memory was assessed by measuring freezing behavior during a 10-min extinction session in Context A. Seventy-four hours after the infusions, long-term memory for the tone-shock association was assessed by measuring freezing during an extinction test in the novel Context B. In this test, an 8-min continuous tone was presented 2 min after a rat was placed in the chamber.

*Tone fear.* Specific associations between cues and fear are formed in the BLA [11]. We used a standard tone-fear conditioning protocol, in which a single tone cue is paired with shock. Detailed surgical procedures were described [50]. Training and testing sessions were conducted in two contextually distinct, aluminum and Plexiglas conditioning chambers, similar to the ones used for contextual fear conditioning. Freezing was automatically measured for 2 s of every 5 s for 10 min (Med PC version 4; Med Associates).

After 3 d of habituation to the two contexts, the rats were placed in Chamber A, and after 4 min, a 30-s, 90-dB, 5-kHz tone was played. The tone coterminated with a 1.5-mA, 1-s foot shock. Thirty seconds later, the rat was returned to its home cage. One hour after exposure to shock in Chamber A, the rat was placed in Chamber B for 5 min without the tone or shock. Twenty-two hours after training, the rats were infused with saline, scr-ZIP, or ZIP. Two or 24 hours later, long-term memory retention was tested by placing the rat in Chamber B, exposing the rats to the tone, and then measuring freezing in response to the tone. Six weeks after tone-shock conditioning, immediate postshock freezing was assessed in a counter-balanced subset of rats (*n* = 18). ZIP or saline was infused into both BLA sites, 5 min or 2 h prior to testing. An animal was then placed in Chamber A, and after 4.5 min, it was shocked once (1.5 mA, 1 s). Freezing behavior was scored during the subsequent 10 min.

### Inhibitory avoidance.

The BLA is important for inhibitory avoidance, because it modulates the strength of information as it is stored at extra-amygdala sites during a posttraining consolidation window lasting several hours [32,33]. Whether the BLA also stores associations for maintaining inhibitory avoidance beyond this time window, however, is controversial. We used a standard inhibitory avoidance protocol in which the rat is shocked once when it enters from the brightly lit side to the dark compartment of the conditioning environment [51].

Experiments were performed as previously described [51]. Briefly, the inhibitory avoidance training apparatus consisted of a rectangular box comprising two compartments, a safe (brightly lit) one and a shock (dark) one, separated by a vertically sliding door (Med Associates). During training, each rat was placed in the safe compartment with its head facing away from the door. After 10 s, the door automatically opened, allowing access to the shock compartment, and latency to enter was taken as a measure of inhibitory avoidance acquisition. The door closed after the rat completely entered the shock compartment, and 2 s later, a brief foot shock (0.9 mA, 2 s) was delivered. The rat was then removed from the apparatus and returned to its home cage. Twenty-two hours after training, the rats were infused with saline, scr-ZIP, or ZIP. Long-term inhibitory avoidance memory was then tested 2 h later, by placing the rat back in the safe compartment and measuring the latency to enter the shock compartment. A foot shock was not delivered during the retention test. For animals that did not enter the shock compartment, the test was terminated at 9 min.

### Statistical analyses.

Group comparisons were made by ANOVA. Significance was accepted for *p* < 0.05. When appropriate, Newman-Keuls post hoc tests were performed. Student *t*-tests were used to compare group performance during retention of place avoidance against the chance value.

## Abbreviations

BLA: basolateral amygdala
DH: dorsal hippocampus
LTP: long-term potentiation
PKC: protein kinase C
PKMζ: protein kinase Mzeta
scr-ZIP: a scrambled, inactive version of zeta inhibitory peptide
ZIP: zeta inhibitory peptide
